# Cyanobacteria newly isolated from marine volcanic seeps display rapid sinking and robust, high-density growth

**DOI:** 10.1128/aem.00841-24

**Published:** 2024-10-29

**Authors:** Max G. Schubert, Tzu-Chieh Tang, Isabella M. Goodchild-Michelman, Krista A. Ryon, James R. Henriksen, Theodore Chavkin, Yanqi Wu, Teemu P. Miettinen, Stefanie Van Wychen, Lukas R. Dahlin, Davide Spatafora, Gabriele Turco, Michael T. Guarnieri, Scott R. Manalis, John Kowitz, Elizabeth C. Hann, Raja Dhir, Paola Quatrini, Christopher E. Mason, George M. Church, Marco Milazzo, Braden T. Tierney

**Affiliations:** 1 Two Frontiers Project, Fort Collins, Colorado, USA; 2 Wyss Institute of Biologically-Inspired Engineering, Boston, Massachusetts, USA; 3 Department of Physiology and Biophysics, Weill Cornell Medical College, New York, New York, USA; 4 Natural Resource Ecology Laboratory, Colorado State University, Fort Collins, Colorado, USA; 5 Department of Chemical and Biological Engineering, University of Wisconsin-Madison, Madison, Wisconsin, USA; 6 Koch Institute for Integrative Cancer Research, Massachusetts Institute of Technology, Cambridge, Massachusetts, USA; 7 Renewable Resources and Enabling Sciences Center, National Renewable Energy Laboratory, Golden, Colorado, USA; 8 Biosciences Center, National Renewable Energy Laboratory, Golden, Colorado, USA; 9 Department of Integrative Marine Ecology, Sicily, Stazione Zoologica Anton Dohrn, Lungomare Cristoforo Colombo (complesso Roosevelt), Palermo, Italy; 10 National Biodiversity Future Center, Palermo, Italy; 11 Department of Earth and Marine Sciences, University of Palermo, Palermo, Italy; 12 Department of Biological Engineering, Massachusetts Institute of Technology, Cambridge, Massachusetts, USA; 13 Department of Mechanical Engineering, Massachusetts Institute of Technology, Cambridge, Massachusetts, USA; 14 Seed Health, Venice, California, USA; 15 Department of Biological, Chemical and Pharmaceutical Sciences and Technologies, University of Palermo, Palermo, Italy; 16 Department of Genetics, Harvard Medical School, Boston, Massachusetts, USA; University of Illinois Urbana-Champaign, Urbana, Illinois, USA

**Keywords:** cyanobacteria, photosynthesis, microbiology, algae, carbon sequestration, microbial diversity, CO_2_

## Abstract

Cyanobacteria are photosynthetic organisms that play important roles in carbon cycling and are promising bioproduction chassis. Here, we isolate two novel cyanobacteria with 4.6Mbp genomes, UTEX 3221 and UTEX 3222, from a unique marine environment with naturally elevated CO₂. We describe complete genome sequences for both isolates and, focusing on UTEX 3222 due to its planktonic growth in liquid, characterize biotechnologically relevant growth and biomass characteristics. UTEX 3222 outpaces other fast-growing model strains on a solid medium. It can double every 2.35 hours in a liquid medium and grows to high density (>31 g/L biomass dry weight) in batch culture, nearly double that of *Synechococcus* sp. PCC 11901, whose high-density growth was recently reported. In addition, UTEX 3222 sinks readily, settling more quickly than other fast-growing strains, suggesting favorable economics of harvesting UTEX 3222 biomass. These traits may make UTEX 3222 a compelling choice for marine carbon dioxide removal (CDR) and photosynthetic bioproduction from CO₂. Overall, we find that bio-prospecting in environments with naturally elevated CO₂ may uncover novel CO₂-metabolizing organisms with unique characteristics.

**IMPORTANCE:**

Cyanobacteria provide a potential avenue for both biomanufacturing and combatting climate change via high-efficiency photosynthetic carbon sequestration. This study identifies novel photosynthetic organisms isolated from a unique geochemical environment and describes their genomes, growth behavior in culture, and biochemical composition. These cyanobacteria appear to make a tractable research model, and cultures are made publicly available alongside information about their culture and maintenance. Application of these organisms to carbon sequestration and/or biomanufacturing is discussed, including unusual, rapid settling characteristics of the strains relevant to scaled culture.

## INTRODUCTION

Cyanobacteria serve as promising hosts for photosynthetic bioproduction (1–4). They carry out oxygenic photosynthesis, which is widely understood to be the most important metabolic innovation in Earth’s history (5). Using CO₂ as a carbon source, light as an energy source, and water as an electron donor, they build complex living materials, converting ubiquitous materials to diverse substrates.

Cyanobacteria potentially enable a variety of new carbon-negative technologies (6–8). Most cyanobacterial research is conducted in model organisms isolated more than 50 years ago (9, 10), but more recently isolated strains from diverse environments demonstrate unique traits and potentially improved biotechnological potential. For example, UTEX 2973 is a spontaneous mutant of the historical type strain PCC 6301 (11), exhibiting high light tolerance and exceptional doubling times as fast as every 1.5 hours (12–14). PCC 11801 and PCC 11802 were isolated from a eutrophic urban lake and displayed fast growth and promising metabolic traits (15–17). PCC 11901, isolated from a fish farm, displays fast growth to unusually high biomass density (18, 19). Cyanobacteria isolated from alkaline soda lakes grow well at high pH, which is beneficial for CO_2_ transfer into water (20, 21).

We hypothesized that due to their exposure to sunlight and high ambient dissolved inorganic carbon, the shallow volcanic seeps off the coast of Baia di Levante in Vulcano Island, Italy, may be rich in biotechnologically relevant cyanobacterial life. This volcanic region features marine volcanic seeps, which continuously release CO₂, and it is actively investigated as a model of ocean acidification and ecosystem structure (22). These shallow seeps are at 1–4 m depth, discharging ~1,300 tons/year (23). For comparison, the few existing flux estimates for volcanic CO_2_ seeps worldwide range from <1 to >2,000,000 tons/year (24). The CO_2_-rich emissions at Baia di Levante result in acidic conditions (<6.5 pH) in the seawater column around the main venting area. The discharged fluids consist of hydrothermal gasses containing elevated CO_2_ (>98%), H_2_S (400 ppm), and CH_4_ (400 ppm) concentrations. The interaction of reduced gasses with seawater leads to dissolved oxygen consumption and reducing conditions (low redox potential, Eh) in seawater (25, 26). Such reducing conditions are also caused by the discharge into seawater of saline hydrothermal brines (derived from a shallow aquifer). These fluids are rich in metals such as iron, whose oxidation leads to extensive oxygen consumption (25, 26). Iron concentrations around the main degassing area are roughly 2–3 times higher than that in control waters (27).

In contrast to the deeper oceanic vents that sunlight does not reach, these shallow seeps have water, light, and CO₂—all crucial for oxygenic photosynthesis—in copious abundance. Carbon is often a limiting factor in cyanobacterial growth in the environment, and high-affinity cyanobacterial carbon concentration mechanisms have evolved to mitigate this limitation (28). We hypothesized that organisms relieved from carbon limitation would potentially realize greater fitness improvement and thus evolve more readily, adaptations addressing other limitations. These could include efficient light utilization, rapid growth and division, evasion of predators, antagonism toward competitors, or countless other possibilities. By isolating organisms from this unique environment, we can expect to discover unique organisms valuable for research and capable of sequestering carbon with high efficiency.

In this work, we endeavored to isolate such organisms, with a focus on cyanobacteria, to contribute to the growing set of novel, promising cyanobacterial hosts for bioproduction. Here, we describe the isolation, observation, and genome sequencing of two related strains found in this unique environment. We additionally describe the initial growth, settling, and biomass characterization of one of these strains, emphasizing how these initial results are early indicators of its potential application as a bioproduction chassis.

## RESULTS

### Obtaining and sequencing fast-growing cyanobacteria

**Fig 1:**
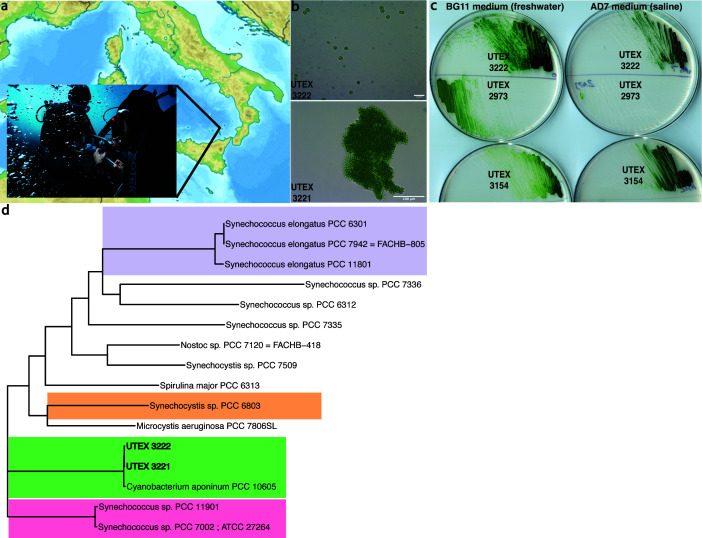
Isolation and sequencing. (a) Samples were obtained from Baia di Levante, Vulcano, Italy. The map was adapted from Wikimedia under the Creative Commons Attribution-Share Alike 3.0 Unported license, https://commons.wikimedia.org/w/index.php?curid=21003231. (b) Micrographs of UTEX 3221 and UTEX 3222, displaying planktonic vs. aggregate growth. (c) Axenic isolate UTEX 3222 grow alongside UTEX 2973 and UTEX 3154 (a derivative of PCC 11901) after 3 days at 37°C, 200 µE, and 0.5% CO_2_. BG11 medium supplemented with vitamin B-12. (d) Phylogenetic tree (using the Bac120 marker genes from the Genome Taxonomy Database) of novel isolates alongside their closest sequenced relative and notable model cyanobacteria. Notable clades are highlighted in color.

We conducted a sampling (Fig. 1a) expedition to the CO_2_-enriched Baia di Levante, near Vulcano Island (Aeolian archipelago, Italy). The CO_2_ in the bay generated from a shallow main venting area (latitude, longitude 38.418722N, 14.963879E, 1–4 m depth), with seawater pH of approximately 6.5. Seawater and sediment temperatures were approximately 25°C and 45°C, respectively, and the maximum pCO_2_ measured in the venting sites was 28,800 µatm (Data S1). Seawater pH and pCO_2_ reached ambient levels (i.e., around 8.1 pH and 400 µatm) at a distance of approximately 500–600 meters from the sites under the most intense CO_2_ leakage (Data S1).

Sediment, water, and biomass were obtained for downstream microbiological analysis on open-circuit scuba from along the entirety of Baia di Levante’s CO_2_ gradient (Fig. 1a, Materials and Methods). This study aimed to isolate fast-growing cyanobacteria from these samples. Multiple seawater and sediment samples were pooled from each dive site, concentrated by filtration, enriched in conditions expected to promote fast liquid growth of phototrophs, and rendered axenic on solid medium (Materials and Methods). Two promising cyanobacterial isolates were obtained from the shallow CO_2_ seep area of Baia di Levante and are now publicly available as UTEX 3221 and UTEX 3222. Both strains grew well on solid medium, creating visible colonies in 2 days of incubation in the conditions described (Materials and Methods). In liquid medium, UTEX 3221 formed macroscopic aggregates of cells across all media and conditions tested, whereas UTEX 3222 exhibited unicellular, planktonic growth (Fig. 1b; Fig. S1A). UTEX 3221 additionally exhibited phototactic motility, which appeared to be absent in UTEX 3222 (Fig. S1B).

We chose to focus on UTEX 3222 for further characterization, as planktonic growth is better explored in the existing literature and presumed to make better use of incident light. UTEX 3222 produced larger colonies than notable fast-growing cyanobacterial model strains UTEX 2973 or UTEX 3154 (PCC 11901 adapted to the absence of vitamin B-12) (19) after 3 days of growth in the conditions tested (Fig. 1c, quantification in Fig. S2), enriched with 0.5% CO_2_. Growth on either BG-11 freshwater or AD7 saline medium suggests that these strains are euryhaline. These isolates do not require vitamin B-12, which is required by PCC 7002, PCC 11901 (18), and, to a lesser extent, UTEX 3154(19). Both novel isolates exhibited an approximate 3.72 ± 0.06 µm diameter spherical cell size, larger than described for other model unicellular cyanobacteria (12, 18, 29).

### Genome characteristics

Genome sequencing and assembly (see Materials and Methods) revealed approximately 4.4 Mbp genomes, and annotation identified coding regions, CRISPR elements, and other elements of interest (Table 1). UTEX 3221 and UTEX 3222 are closely related strains, sharing more than 98% average nucleotide identity (ANI) and differing by at least two major genome inversion/translocation events (Fig. S3). Phylogenetic comparison reveals the closest known sequenced relative of these strains to be *Cyanobacterium aponinum* PCC 10605 and confirms that these strains reside apart from clades containing highly-studied cyanobacterial model strains (Fig. 1d; Fig. S4). Comparison of genomic regions by BLAST revealed genomic blocks with lower similarity among comparison strains, potentially highlighting novel elements in these genomes. antiSMASH(30) identified biosynthetic clusters of interest, including terpenes, arylpolyene, lanthipeptides, and an iucA/iucC-like siderophore. Notably, PCC 10605 also exhibits these pathways.

**TABLE 1:** Summary of genome characteristics^*a*^

	UTEX 3221	UTEX 3222	PCC 10605
Chromosome	4,417,179 bp	4,280,321 bp	4,114,099 bp
Assembly status	Circular	Circular	Circular
Episomes	None detected	None detected	62,874 bp circular
Transfer RNA	44	45	44
Transfer-messenger RNA	1	1	1
Ribosomal RNA	9	9	9
Non-coding RNA (ncRNA)	9	9	9
Regions regulating ncRNA activity	5	5	5
CRISPR	7	6	11
Coding sequences (CDS)	3,760	3,602	3,480
Small open reading frame (sORF)	1	1	0
Origin of replication (oriC)	1	1	0
PhiSPY prophage candidates	2	2	1
Average nucleotide identity (ANI) to PCC 10605 genome	98.41%	98.45%	100%

^*a*^ Annotations produced by annotation with BAKTA (31), Prophage detection with PhiSPY (32) using the PHROG database (33), and average nucleotide identity (ANI) using FastANI (34).

This close relative, PCC 10605, was also isolated in Italy, from thermal springs (35), and has been studied with regard to DNA replication (36) and C-phycocyanin production (37). Other relatives of PCC 10605 have been isolated worldwide, including from a marine environment near hot springs in China (38) and a wastewater treatment system in Oman (39). PCC 10605 did not appear to grow as quickly as our isolates on solid medium in the conditions tested (Fig. S5) and displayed an aggregation phenotype in liquid similar to UTEX 3221.

UTEX 3221 and UTEX 3222 thus display intriguing fast growth and genomic features and belong to a clade of cyanobacteria containing *Cyanobacterium aponinum* PCC 10605 and related isolates. There is not extensive research on this clade, particularly concerning the investigation of fast-growing isolates and their application as chassis strains in synthetic biology.

### Growth characterization of UTEX 3222

**Fig 2:**
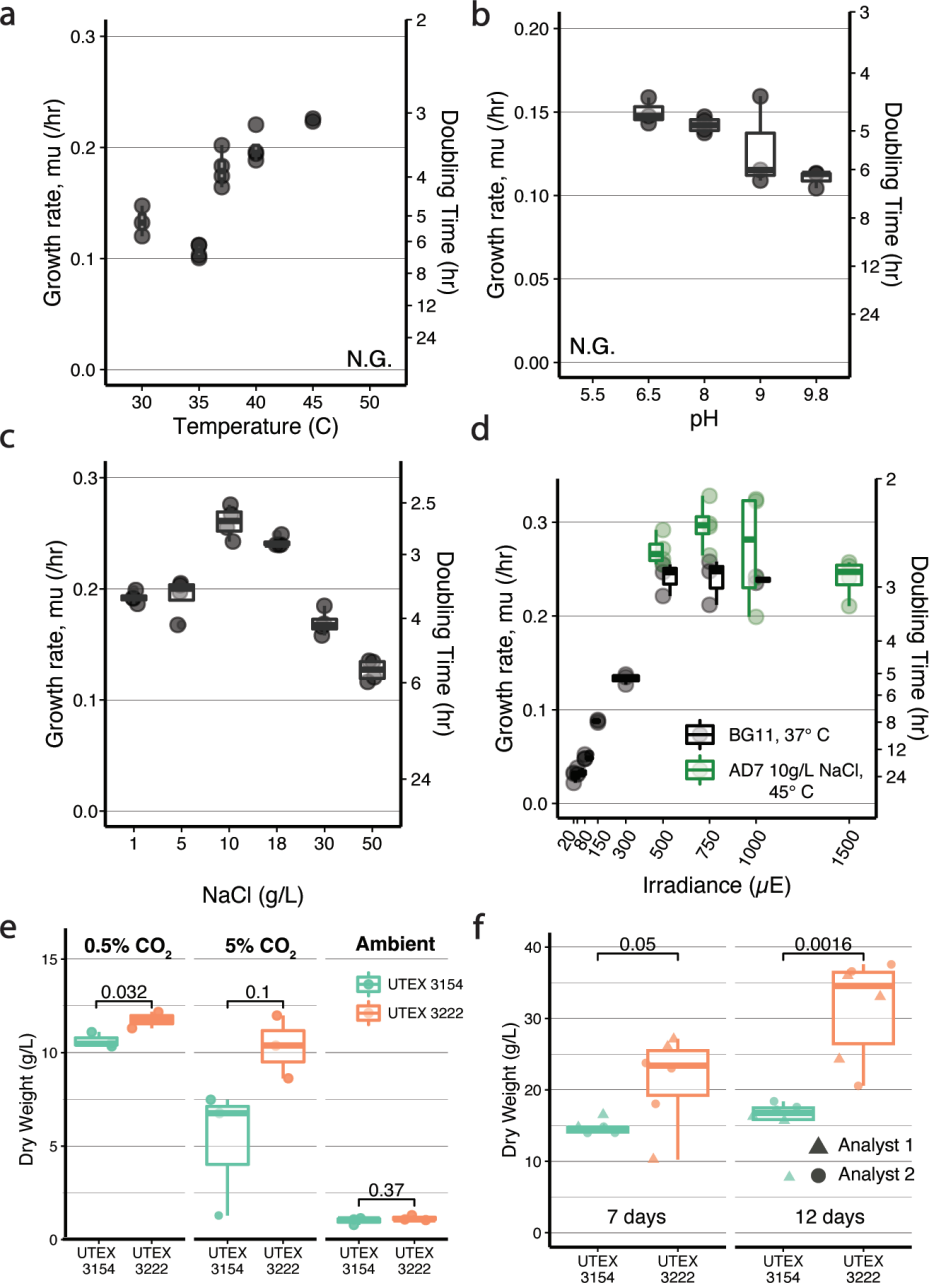
Growth conditions and high-density growth. Exponential growth rate of UTEX 3222 in BG11 medium at varying temperature (**a**), pH (**b**), salt concentration (**c**), and total light (**d**) in the multicultivator instrument. No growth (NG) was observed at 50°C or at pH 5.5. Where otherwise not noted, liquid growth conditions were BG11 medium at 37°C 500 µE of light, pH 8.2, and 0.5% CO_2_. Panel C details growth trials across salt concentration, using a modified AD7 saltwater medium (see Materials and Methods). Optimal temperature, salt, and media conditions were compared with the standard BG11 37°C growth condition across a range of irradiance in panel D. (**e**) Biomass dry weights (gDW/L) after 7-day batch incubation varying CO_2,_ using flasks at 200 µE light, 37°C, MAD2 medium (Materials and Methods). (**f**) Biomass dry weight after 7 and 12 days with light increased to 750 µE on day 2, 1% CO_2_, 37°C, MAD2. Individual replicates are depicted with circles or triangles (indicating independent experiments conducted by two authors).

We next completed pilot experiments to evaluate the growth of UTEX 3222 in liquid media, measuring the exponential growth rate after dilution to an OD of 0.1 (see Materials and Methods). We began with growth in BG-11 freshwater medium, where UTEX 3222 produced the most robust growth on solid medium (Fig. 1b). In BG-11, UTEX 3222 displayed a wide tolerance to temperature, with an optimal growth rate at 45°C among the conditions tested (Fig. 2a), somewhat higher than 30°C or 37°C conditions used for most model cyanobacteria (14, 18). This temperature optimum is higher than previous reports in this clade of cyanobacteria (35, 38) and reflects realistic temperatures observed in outdoor photobioreactors midday in the absence of cooling equipment (40, 41). At the same time, the growth rate appears faster than thermophilic models like *T. elongatus* BP-1, isolated from hot spring environments, which has a higher temperature optimum of ~57°C (42), indicating the utility of volcanic seeps for new biotechnological chassis discovery.

Although noting this optimum at 45°C, we continued at 37°C when exploring other variables to ease comparison with outside literature and isolates, such as UTEX 2973, which is often characterized at 38°C (12, 43, 44), and PCC11901, which is also often characterized at 38°C (19). No growth was observed at pH 5.5, but pH from 6.5 to 9.8 was well-tolerated (Fig. 2b). The fastest exponential growth was observed at pH 6.5, identical to the isolation site, but higher cell density was quickly achieved at pH 8, likely due to better availability of bicarbonate at this pH (Fig. S6B). Commensurate with its marine habitat, UTEX 3222 tolerated high levels of salt, even exceeding that of seawater, although a moderate 10 g/L NaCl produced the fastest growth (Fig. 2c). Notably, this differs from results on solid medium, where BG-11 medium appeared to promote faster growth (Fig. 1b). We did not observe elongated cells in elevated salinity, a phenotype occurring in the close relative PCC 10605 (35).

UTEX 3222 tolerated irradiance of at least 1,500 µE (Fig. 2d), which is lethal for most photosynthetic microbes at these culture densities but tolerated by some fast-growing isolates (45). In BG-11 medium at 37°C, growth rates increased with increasing light up to 500 µE, beyond which more light did not produce a higher growth rate. Combining the optimal temperature, pH, and medium/salinity results from previous experiments supported a faster growth rate overall, up to 750 µE with doubling times of 2.35 ± 0.10 hours (Fig. 2d). It is likely that additional optimization could result in faster exponential growth, but we chose to instead focus next on high-density growth.

### High-density growth of UTEX 3222

Although fast growth in solid medium (Fig. 1b) and short exponential doubling time in liquid (Fig. 2a through d) are good predictors of an organism’s facility in the lab and preferences among growth conditions, light-limited growth at high density is predicted to be more relevant for industrial applications, where high culture density drives higher volumetric or areal productivity (46, 47). Following an initial observation of planktonic liquid growth of UTEX 3222 to high density, we explored high-density batch growth as a relevant industrial behavior.

Recent work reports record-setting cyanobacterial culture densities in PCC 11901 (18), and indeed, high-density growth appears to be a unique characteristic of this strain. Further development of PCC 11901 yielded UTEX 3154, a derivative with an alleviated vitamin B-12 requirement (19). To explore high-density growth in UTEX 3222, we thus used MAD2 saline medium and antifoam providing the highest biomass yield in PCC 11901 (18) (Materials and Methods) for all high-density measurements and grew alongside UTEX 3154 as a control and comparison. Beginning with MAD2 medium in our original culture enrichment conditions (0.5% CO_2_ 200 µE light, 37C), which appear to be reasonable conditions for comparing the two strains (18, 19), we found that UTEX 3222 grew to high density, even surpassing that of UTEX 3154 in these conditions by optical density over time (Fig. S7A) and by biomass dry weight at 7 days (Fig. 2e, 0.5% CO_2_).

To explore high-density growth across varying conditions, we first varied the growth chamber CO_2_ concentration (beginning with our initial enrichment conditions). Growth in high CO_2_ has been explored for cultivation on flue gas or other point source emissions but is often not well-tolerated by algae and cyanobacteria (48–50). Conversely, ambient air contains low CO2 (~400 ppm) and can be limiting for photosynthetic growth (20, 21). Raising CO_2_ to 5% decreased the dry weight of both strains, and the use of ambient air conditions (~0.04% CO_2_) predictably led to far lower dry weight in both strains (Fig. 2e). Increasing salt concentration of MAD2 (18 g/L) to more closely approximate seawater (30 g/L) again led to significantly higher biomass in UTEX 3222 and appeared to increase dry weight in both strains, but not by a substantial margin(Fig. S7B). Increasing the initial inoculum also did not meaningfully raise the biomass titer (Fig. S7C), suggesting that batch growth was saturated after 7 days in these conditions. We thus next explored conditions that provided the highest biomass yields in prior work (18), increasing light to 750 µE after 1 day and using 1% CO and MAD2 medium. These conditions produced higher density still (Fig. 2f), with UTEX 3222 yielding 31.36 ± 2.92 g/L biomass after 12 days and UTEX 3154 yielded 15.49 ± 1.59 g/L. Further work to compare with other model cyanobacteria and optimize medium and conditions specifically for UTEX 3222 or conduct continuous culture at high density could potentially increase productivity even further.

### Biomass composition of UTEX 3222

**Fig 3:**
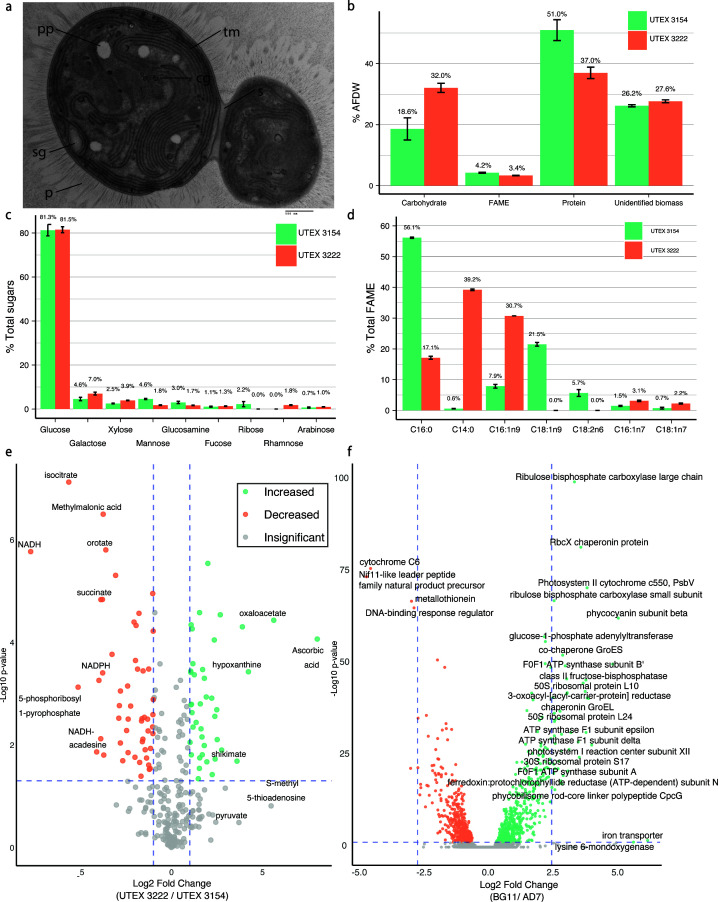
Characterizing UTEX 3222 biomass. (a) Representative TEM image, with abundant thylakoid membrane (tm), division septa (S), and putative polyphosphate granules (pp), storage granules (sg), cyanophycin granules (cg), and extracellular pili (P). (**b**) Major macromolecule composition, as a percentage of ash-free dry weight (% AFDW). (**c**) Sugars were detected following acid hydrolysis of biomass. (**d**) Fatty acid methyl ester (FAME) species, species with a mean measurement >2% shown, for lower abundance species see Fig. S10. For all charts in this figure, bars depict the mean of triplicate culture measurements, and error bars depict the standard error of these measurements. (**e**) Relative comparison of a panel of polar metabolites across UTEX 3222 and UTEX 3154 biomass grown to high density. Analytes differing in abundance by more than 10-fold are labeled with text. (**f**) Differential expression analysis by RNA-sequencing of UTEX 3222, comparing freshwater (BG-11) growth with saline (AD7) medium.

Because UTEX 3222 is not closely related to well-studied model cyanobacteria (Fig. 1c), we expected its cellular composition to differ. These differences could inform efforts to use the cyanobacterial biomass itself or potentially engineer this organism to produce new products. We began by exploring cellular composition qualitatively by transmission electron microscopy (TEM) of cells from high-density culture, revealing putative extracellular polysaccharides (EPS), as well as what appeared to be storage granules for glycogen or polyhydroxyalkanoates, both common carbon storage products in cyanobacteria (Fig. 3a; Fig. S7A, B, and D) (51). Relevant biosynthetic genes for both products are found in the UTEX 3222 genome. Further images reveal heterogeneity; cells grown in the conditions tested appear to vary in the number and size of storage granules (Fig. S8). UTEX 3154 grown and imaged in a similar manner did not display as prominent EPS or storage granules (Fig S8E and F).

Quantitative analysis of overall biomass composition revealed a higher proportion of carbohydrates in high-density UTEX 3222 biomass compared with UTEX 3154, with correspondingly lower fractions of ash-free dry weight consisting of protein and other components (Fig. 3b; Fig. S9A). This overall difference was also observed in C/H/N elemental analysis, with UTEX 3222 biomass having higher overall carbon content (Fig. S9B). These measurements argue that UTEX 3222 may produce more storage carbohydrates than UTEX 3154 when grown to high density. This could explain in part differences in the biomass density achieved in identical conditions.

**Fig 4:**
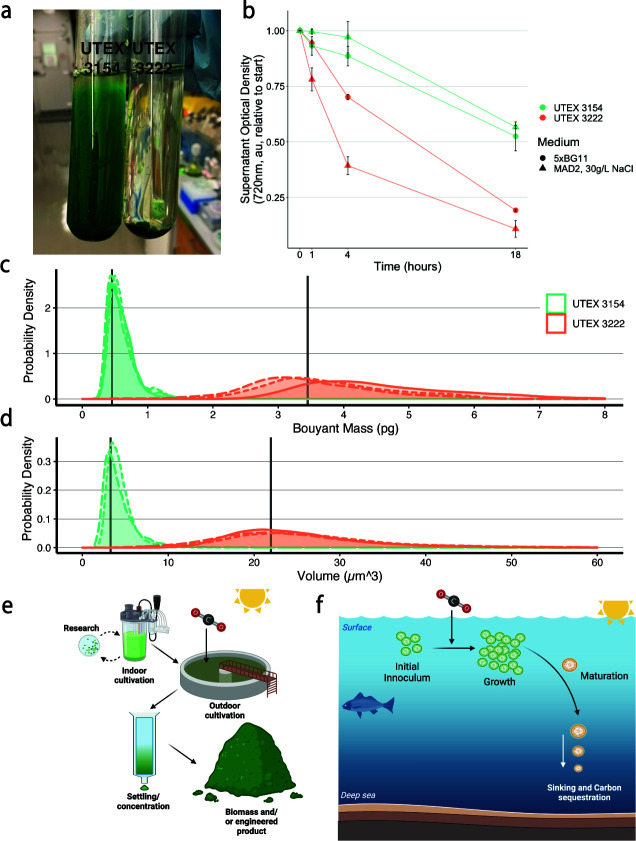
Sinking behavior of UTEX 3222. (a) Photograph of cultures was allowed to settle for 12 hours at 20°C, after 12 hours of growth at 37°C in BG11 medium. (b) Sinking timecourse, employed with high-density batch-cultivated cultures at 20°C in the absence of light. Lines and points display the mean of triplicate experiments; error bars are standard errors. (c) Buoyant masses of single cells, as measured using the SMR (see Materials and Methods). (d) Volumes of single cells, as measured using the CC (see Materials and Methods). Data in C and D are presented as a probability density function with individual replicate experiments plotted separately with different line styles. In C, the difference between replicates reflects biological variance in cells’ buoyant density. The peak of this density function approximates the mode of data, and the mean of these peaks is shown. (e) Schematic summarizing that these strains support rapid research and development due to their fast growth rates in the lab and that their facile sinking phenotypes could be leveraged to improve production of biomass and/or engineered products at scale. (f) Schematic summarizing a potential marine carbon sequestration procedure, in which sinking phytoplankton preferentially support movement of carbon into deep ocean sediments.

Acid hydrolysis of carbohydrates derived from either strain reveals the overall composition of component sugars, with >80% of carbohydrates in both strains digesting to glucose but significant differences in lower-abundance sugars (Fig. 3c). In particular, where rhamnose comprises nearly 2% of hydrolyzed sugars from UTEX 3222, it is undetectable in UTEX 3154, and in contrast, ribose was not detected in UTEX 3222. Notably, both strains contain relatively low concentrations of fatty acid methyl ester lipids (FAME, Fig. 4b; Fig. S10), which can be quite abundant in oleaginous model phototrophs. The differing composition of these FAME across the two strains was notable; however. UTEX 3154 biomass contained far more palmitic acid (C16:0), as well as abundant oleic acid (C18:1n9) and linoleic acid (C18:2n6), which were both undetectable in UTEX 3222 (Fig. 4d). Conversely, the FAME in UTEX 3222 contained far more myristic acid (C14:0) and hypogeic acid (C16:1n9) (Fig. 3d) and had overall shorter FAME chain lengths.

Similarly, metabolites can be compared between these strains in order to help understand their differences and characterize UTEX 3222 as a chassis for metabolic engineering and as a source of high-density phototrophic biomass. Mass spectroscopy of UTEX 3222 and UTEX 3154 biomass when grown to high density provides relative quantification of metabolites, and by comparing a targeted panel of 292 common polar metabolites, we see myriad differences emerge (Fig. 3e). UTEX 3222 produces ascorbic acid (vitamin C), which is undetectable in UTEX 3154. Shikimate, a precursor in the biosynthesis of aromatic compounds, is elevated compared with UTEX 3154. Similarly, mevalonate, a precursor for terpene synthesis, is elevated in comparison to UTEX 3154. These differences were observed when growing strains in identical medium and conditions; further characterization across multiple conditions and physiological states would provide additional context. For instance, NADPH and NADH appear more abundant in UTEX 3154, but these energy carriers are more likely to change quickly depending on the physiological state or extraction procedure (52), and quantification in such an assay is less confident.

Such physiological differences likely vary across culture conditions, and transcriptomics helps to more mechanistically describe these physiological states as well as further elucidate genome annotation. We thus interrogated the transcriptome of UTEX3222 when grown in either AD7 saline medium or BG11 freshwater medium by strand-specific RNA sequencing. As expected, the two media yielded dramatically different expression profiles. One thousand twenty-six genes were significantly (adjusted *P*-value < 0.05) increased in expression in BG11, whereas 948 increased in AD7 medium (Fig. 3f; Data S2). This corresponds to 52% of coding frames in the genome having significant variation in expression.

Genes associated with growth and central metabolism (e.g., RuBisCO, light harvesting proteins like PsaB, PsaC, and PsbH) were increased in expression in BG11 relative to AD7. We also identified potential responses to iron starvation in BG11 (with increases in genes associated with iron acquisition and transport, e.g., PsaC, IscA, Fdx, SdhB, FrdB, QcrA, PetC, BchL, among others), highlighting that common media might limit growth, even for low-density batch cultures. Iron starvation specifically may be explained by the high iron in the native Baia di Levante habitat. Overall, transcriptomics proved valuable for understanding responses to and potentially optimizing growth conditions.

### Sinking/settling phenotype

In the routine handling of UTEX 3222, we observed that liquid cultures settled into a tight pellet after several hours without agitation, whereas cultures of other fast-growing strains did not settle as quickly nor completely (Fig. 4a). This differing behavior could potentially be of use for industrial processing, where concentration and dewatering of biomass is a substantial economic challenge (53, 54), estimated to contribute from 15% (55) to as much as 30% of production costs (56). A time course provided a more detailed look at the cell settling of high-density biomass in an artificial seawater medium, confirming that UTEX 3222 biomass settles more quickly than that of the comparison strain UTEX 3154 (Fig. 4b). This is the case whether the high-density culture was grown in saline MAD2 medium or in freshwater 5X BG11 medium, although curiously, culture grown in saline medium appears to settle more quickly (Fig. 4b).

Given UTEX 3222’s lack of observed motility, two differing phenomena could drive this settling behavior, aggregation of single cells into larger particles that sink more readily and/or individual cells having faster settling rates. Aggregation plays a key role in the behavior of fast-settling mutants of PCC 7942 cyanobacteria (57) but can be predicated on the ionic strength of the settling medium and can be mimicked with chemical flocculants (55). We were curious whether individual cells had advantageous settling behavior because this could drive the sinking of biomass in more dilute conditions, such as cells growing in marine habitats and affecting natural carbon cycles (58). The gravitational sinking of a cell, as defined by Stokes’ law, is dependent on cell volume and buoyant density. We measured the buoyant masses and volumes of single cells as previously described (Fig. 4c and d) (58). As buoyant mass is a function of cell volume and cell buoyant density, this allows us to solve for the buoyant densities and, thereby, gravitational sinking velocities of the cells. This revealed that individual cells of UTEX 3222 have 2.16-fold faster predicted gravitational sinking velocity than UTEX 3154 comparison cells (Table 2). This difference in sinking velocity was mediated primarily by greater cell volume rather than differences in buoyant density. Previous results point to starvation responses potentially increasing algal mass and sinking velocity (58), which is in line with the putative storage polymers observed in UTEX 3222 (Fig. S8) and the putative nitrogen starvation response observed in this growth medium (Fig. 3f).

**TABLE 2:** Physical parameters of UTEX 3222 cells drive different sinking behaviors^*a*^

Strain	Replicate	Buoyant mass (pg)	Volume (μm^3^)	Buoyant density (g/mL)	Radius (inferred, μm)	Sinking velocity (μm/s)	90% interval (buoyant mass, ng)	Events observed (mass, SMR)	90% interval (volume, µm^3^)	Events observed (volume, CC)
UTEX 3154	1	0.442	3.709	1.139	0.96	0.233	(0.288–1.201)	2,414	(2.106–8.464)	33,332
UTEX 3154	2	0.496	3.479	1.163	0.94	0.273	(0.289–1.157)	590	(2.09–7.157)	19,621
UTEX 3154	3	0.436	3.021	1.164	0.897	0.263	(0.274–0.978)	548	(1.583–8.323)	15,964
UTEX 3222	1	3.913	21.022	1.206	1.712	0.649	(2.633–7.064)	655	(12.33–41.697)	44,744
UTEX 3222	2	3.296	23.238	1.162	1.77	0.511	(2.123–5.917)	474	(7.136–48.247)	51,942
UTEX 3222	3	3.082	21.567	1.163	1.727	0.502	(1.631–5.877)	622	(7.652–47.184)	56,258

^*a*^ Buoyant masses of cells were measured with a suspended microchannel resonator (SMR), and volumes were measured with a Microsizer or “Coulter Counter” (CC) instrument (see Materials and Methods, Fig. 4c and d). The summary of data is reported alongside the intervals in which 90% of the data are contained, and the number of observations made.

Although UTEX 3222’s derived sinking velocity exceeds that of UTEX 3154 by 2.16-fold, this insufficiently accounts for the stark difference in settling overnight (Fig. 4a), or the >5-fold more settled supernatant after 4 hours (Fig. 4b) This indicates that aggregation or other factors are likely significant drivers of this difference as well. Notably, the sister strain UTEX 3221 and the relative strain PCC 10605 exhibit large aggregates in the conditions tested that settle far more quickly (Fig. S1) and may offer clues for further manipulating aggregation and settling, carrying additional industrial benefit. A better understanding of phytoplankton sinking in seawater also improves our understanding of these organisms' involvement in natural carbon cycles (58, 59) and presents intriguing possibilities wherein sinking behavior could be used to drive stable carbon sequestration in bioreactors (Fig. 4e) and the deep ocean (Fig. 4f).

### Attempted transformation efforts

In a pilot set of experiments, we were unable to transform UTEX 3222 via electroporation, natural competence, or conjugation. We modeled our strategy on existing approaches (see Materials and Methods) that have been successful for other cyanobacteria (60, 61). Ultimately, the three methods we attempted were unsuccessful, failing to yield visible transformed colonies of UTEX 3222. We recommend future work to focus on integrating vectors, where the unknown replication of episomes in UTEX 3222 is not a confounding factor. We also found evidence of abundant restriction/modification and CRISPR systems in our genome annotation, which we hypothesize may serve as barriers to introduced DNA. Notable progress has been made to evade such systems, and the next attempts to engineer UTEX 3222 could leverage these developments (62). We recommend future work in this area address these potential limitations, and our further analysis of restriction/modification systems using REBase (63) aims to support such efforts.

## DISCUSSION

In this study, we identified, sequenced, and characterized UTEX 3222 and UTEX 3221: two photosynthetic fast-growing strains of *Cyanobacterium aponinum* isolated from high CO_2_ marine volcanic seeps in the coastal Mediterranean Sea. UTEX 3221 formed cell aggregates during growth, whereas UTEX 3222 displayed rapid, planktonic growth to high density; given this result, we chose to further investigate UTEX 3222’s potential as a biotechnological chassis. We propose that (i) its rapid growth on solid medium in the lab, makes it an intriguing lab model. (ii) Its rapid growth in liquid culture (as fast as 2.35-hour doubling time in this study), and tolerance of high light, high salinity, and high pH could support biotechnological applications. (iii) Its growth to high density in batch culture (>30 g/L dry weight) could benefit industrial productivity and harvesting. (iv) The composition of its biomass differs from existing model strains, possibly presenting new opportunities for biomass valorization and metabolic engineering, and (v) the sinking/settling behavior of this biomass offers potential benefits for biomass harvesting or carbon dioxide removal and sequestration in marine environments.

We do note, however, that although the growth of UTEX 3222 is impressive in this study, we do not claim it to be outright “superior” to any other potential chassis. This is both because (i) the conditions we used for its evaluation were not meant to be a systematic comparison across strains and (ii) exponential growth rate, in and of itself, is so optimizable and variable across labs that it is not a reasonable metric for evaluating an organisms biotechnological potential. Growth rate measurement in cyanobacteria is also difficult to reproduce between labs, varying as much as 36% even when the same strain and methods are used, likely due to the intrinsic sensitivity to light quality and air composition (56). In this work, we observe both UTEX 3222 producing robust growth on solid medium (Fig. 1b) and also having a high growth rate in conditions optimized for high-density growth. (Fig. 2d) (64). This result is promising and technically 45% faster than UTEX 2973 in the same conditions, but it should be viewed in a broader context, wherein UTEX 2973 is also capable of exemplary exponential growth when growth is optimized with very high light and CO_2_ conditions (1.5-hour doubling, ~57% faster growth rate) (14). Moreover, faster growth rates for other strains have been reported, albeit in differing conditions and methods of measurement (18). Further comparisons of growth rates of different species in varying conditions (e.g., light, CO2, temperature, pH)—perhaps with additional transcriptomics to understand the metabolic shifts associated with these changes—are needed to make any strong claims about the robustness/speed of growth of one strain versus another. As a result, we emphasize here UTEX 3222’s high-density growth and settling, which are potentially of greater biotechnological relevance than growth rate itself (46, 47). We are not aware of another study reporting a cyanobacterial isolate that is capable of robust growth to high density that additionally settles rapidly.

Marine phytoplankton account for about half the photosynthetic primary production on earth (165–183Gt CO_2_/yr) (65, 66), fixing approximately 3-fold as much carbon as total anthropogenic greenhouse gas emissions(59 ± 6.6 CO_2_e/yr) (67). It is estimated that about one-fifth of this carbon captured is exported to the deep ocean (68). Thus, approaches that could meaningfully increase this fraction could have a tremendous impact and are an area of active study (69). The Intergovernmental Panel on Climate Change (IPCC), as well as the National Academies of Sciences, has highlighted the need for negative emissions technologies (or carbon dioxide removal/sequestration) to avoid the worst effects of anthropogenic climate change and ocean acidification (70, 71). These same bodies also acknowledge that we suffer from a shortage of proven solutions to fit this need and recommend developing a variety of approaches to achieve negative emissions. The cyanobacteria isolated here show early-stage potential to help solve longstanding challenges in this area. By accumulating carbon-rich storage polymers internally and carbon-rich EPS externally, such strains could accumulate a very high carbon-to-nutrient ratio. This offers a potential solution to the “nutrient-robbing” problem plaguing the marine biological pump, wherein precious nutrients are co-sequestered along with the carbon contained in phytoplankton biomass (69). In addition, because the most abundant organisms in the ocean are very small cyanobacteria (72), which are expected to have low sinking rates, interventions that shift marine microbial populations toward larger, faster-sinking organisms would be expected to increase the fraction of carbon exported to the deep ocean, rather than cycling back into the atmosphere. The strains described here accumulate carbon-rich storage polymers and sink readily in seawater. Their fast growth in lab conditions facilitates further experimentation toward the development of these kinds of interventions.

The high biomass productivity of UTEX 3222 could make it a valuable resource for carbon dioxide removal (CDR), which is an important tool for the mitigation of anthropogenic climate change (73). With a CO_2_ biofixation rate of approximately 1.7 kg CO_2_ l^−1^ year^−1^ (as determined using data presented in this manuscript and assumptions used to calculate biofixation rates in previous reports (74, 75), it would take less than 13 liters of UTEX 3222 to capture as much CO_2_ annually as a tree (76). Alternatively, it would take almost 30 liters of UTEX 3154 (Fig. S12A).

Assuming (i) its successful scale-up, (ii) growth rate optimization, (iii) the complete burial or conversion of its biomass, and (iv) limited CO_2_ release upon biomass decomposition, UTEX 3222 would outperform the CO_2_ biofixation of trees on an areal basis (Fig. S12B). Although UTEX 3222 has not been tested in outdoor cultivation, we can predict its performance based on other microalgae strains that have been tested both in lab and outdoor cultivation settings. *Spirulina platensis*, a cyanobacteria commonly grown outdoors for bioproduction, can biofix approximately 142t CO_2_ ha^−1^ year^−1^ (77, 78). Assuming the increase of over 70% between the CO_2_ biofixation rates of *Spirulina* and UTEX 3222 grown in controlled laboratory conditions translates to open pond cultivation, we can expect UTEX 3222 to biofix over 2,000t CO_2_ ha^−1^ year^−1^. The forest landscapes with the highest CO_2_ removal rates range from 4.5 to 40.7t CO_2_ ha^−1^ year^−1^ (79). Other microalgae grown in open-pond systems with potential for carbon dioxide removal include *Tetraselmis* with a CO_2_ biofixation rate of 131t CO_2_ ha^−1^ year^−1^ (80), a mix of strains (*Scenedesmus obliquus* UTEX393, *Monoraphidium minutum* 26B-AM, and *Desmodesmus intermedius* C046) were shown to have a CO_2_ biofixation rate of 117t CO_2_ ha^−1^ year^−1^ (81), and *Picochlorum celeri* was shown to have a CO_2_ biofixation rate of 19t CO_2_ ha^−1^ month^−1^ in the most productive month demonstrated, but this number cannot be extrapolated to an annual rate as the strain is not grown outdoors year-round (82). Alternative methods for outdoor cultivation may increase CO_2_ removal but come with additional upfront costs, *Scenedesmus acutus* (UTEX B72) was shown to have a CO_2_ biofixation rate of 214t CO_2_ ha^−1^ year^−1^ when grown in a photobioreactor (83).

In total, our core hypothesis was that shallow carbon dioxide seeps would contain novel organisms for carbon sequestration, and our results indicate that is the case. Therefore, although UTEX 3221 and UTEX 3222 are promising biotechnological chassis, we claim that their isolation only further indicates the potential of searching natural and underexplored environments for novel microbial life and diversity, which in turn mandates effective conservation of these ecosystems (84). Furthermore, culturing rather than sequencing provides advantages in detecting rare organisms below the detection limit of metagenomic sequencing and providing a detailed understanding of the behaviors of specific organisms. Overall, we expect that continued microbial exploration of CO_2_ seeps and other interesting ecosystems will yield further, more optimal organisms for carbon sequestration and other societally important challenges.

## MATERIALS AND METHODS

### Sampling and isolation

Hundreds of samples of seawater and sediment were collected along a well-established pH/*p*CO_2_ gradient in Baia di Levante (Vulcano Island, Italy). To assess the spatial variation in the carbonate chemistry, a Hobo MX2501 submersible pH/Temperature logger and a Hydro II CO_2_ logger (Contros System & Solutions GmbH, Germany) were deployed at 1–4 m depth in each sampling site. Seawater pH (NBS scale) and temperature (T, °C) were recorded at 1-minute intervals. The pH logger was calibrated with standard buffer solutions (for NBS scale) of pH 4.01, 7.00, and 10.00 and then converted to total scale (pHT). The Hydro II CO_2_ logger recorded pCO_2_ (μatm) every 10 seconds. Seawater samples for salinity and total alkalinity (TA) were also collected in triplicate. Seawater was filtered at 0.45 µm using disposable cellulose acetate filters and stored at room temperature in the dark until TA was measured by a titration system (TiTouch i915, Metrohm). The titrations were cross-validated using a working standard (SD: ±9 µmol kg^−1^) and against certified reference material from the A.G. Dickson laboratory. Fifty-milliliter samples were obtained using syringes and kept at ambient temperature in 50 mL conical tubes with exposure to light for several days, then packed and shipped in darkness at approximately 4°C. Seawater pCO_2_ was calculated from pHT, temperature (T; °C), salinity, and total alkalinity (TA; mmol kg−1) using the Carb function (flag = 8) in the seacarb package (Lavigne and Gattuso, 2010) in RStudio software (version 4.2.1) (85, 86).

Multiple samples per dive site were pooled by filtering using a sterile 0.22 µM filter and then rinsing material from the filter using 50 mL of Enrichment medium. Enrichment medium (AD7++) was AD7 medium prepared as per Włodarczyk et al. (18) and modified by including 100 mg/L of cycloheximide to inhibit the growth of eukaryotes, addition of ATCC trace mineral supplement (MD-TMS, ATCC) and ATCC vitamin supplement (MD-VS, ATCC) at 1/200th strength, and substitution of Tris-HCl for 10 mM of TES-KOH pH 8.2. After initial enrichment, routine culture was performed in AD7 medium or BG-11 medium, also buffered with TES-KOH pH 8.2. Enrichment cultures were cultivated in 250 mL baffled glass Erlenmeyer flasks, closed by both foam stoppers and clear plastic flask closures. Cultures were incubated at 0.5% CO_2_, with 200 μE of white LED light at 37°C in an illuminated incubator (Multitron, InforsHT) shaking at 220 rpm. For some enrichments, visible white growth occurred after 1–2 days. This growth was likely from iron-oxidizing bacteria due to iron present in sediment samples. Such enrichments were subjected to a further 100-fold dilution in AD7++, and in some cases resulted in green, photosynthetic unicellular growth after 6 days in these conditions. Some enrichments yielded macroscopic green “spidery” growth, which appeared to be filamentous cyanobacteria, which proved difficult to render axenic.

Samples from two enrichments exhibiting unicellular green growth were streaked on plates of AD7++ solidified with 1% agar (bacteriological grade, APEX). These were incubated at 0.5% CO_2_, with 200 μE of Percival SciWhite LED light at 37°C in a growth chamber (AL-41L4, Percival). Green colonies were observed alongside white colonies, presumed to be heterotrophs subsisting on agar, buffer, or cyanobacterial exudate. Green colonies were restreaked to solid AD7++ after 3–4 days, and this procedure was repeated at least five times with the goal of obtaining axenic cyanobacterial cultures. Multiple isolates from each enrichment were rendered axenic, but in all cases, these were later determined to be identical strains. Filamentous cyanobacteria were plated to a solid medium but proved difficult to separate from non-photosynthetic contaminants and were excluded from further characterization. Photos on solid medium were obtained on a flat-bed scanner (Epson), and colony size was measured by image analysis in Fiji (87).

### Culture, cryo-preservation, and phototaxis assay

Routine growth of liquid cultures was performed by culturing a 10 mL volume in 50 mL Erlenmeyer flasks in either AD7 or BG-11 medium buffered to pH 8.2 using 10 mM TES-KOH, at 0.5% CO_2_, with 200 μE of white LED light at 37°C, shaking at 120 RPM. We recommend these conditions; while noting that optimal growth is likely obtained in AD7 medium modified to 10 g/L NaCl, 750 µE light, and 45°C, Cryo-preservation was performed by the addition of DMSO to 9%, flash-freezing with liquid nitrogen, and storing at −80°C. Frozen cultures are revived by streaking to AD7 or BG-11 medium and incubating as above, but with 100 μE light and ambient air for the first day and then as above for subsequent days. Cryo-preserved cultures remain viable in this way for more than 18 months and likely longer. Incubator temperatures were confirmed with a NIST-traceable thermometer (Digi-Sense), and photosynthetically active light was confirmed with a light meter (MQ-500, Apogee).

Light microscopy of wet-mounted liquid cultures was performed using a Zeiss Axio Imager Z-1 microscope, illuminated by a white LED light source. Cell size was measured with image analysis in FIJI (data in Data S3). Phototactic motility was investigated by adapting published methods (88). Briefly, liquid culture was plated on BG11 with 0.3% agar (“swim medium”), and plates were incubated in the Percival incubator as described above, but in a foil packet to ensure light only entered from one side, for 4 days. Limited light available inside the foil packet likely resulted in slower growth overall.

### Genome sequencing and analysis

Cultures were grown as above, and 5 mL of overnight culture was pelleted at 4,000 Relative Centrifugal Field (rcf) and stored at −20°C. Light microscopy (Fig. 1b; Fig. S1) and lack of observed growth on LB medium suggested isolates were likely axenic, an assertion supported by the lack of contaminating sub-assemblies after DNA assembly. DNA was extracted from frozen pellets using the ZymoBIOMICS DNA Miniprep Kit (Zymo, Cat. # R2002), performing the initial bead disruption step using a Tissuelyzer LT (Qiagen, Cat. # 85600) set to 50 Hz for 30 minutes, at 4°C. DNA was quantified using the Qubit 1xds DNA, broad range assay (ThermoFisher Scientific, Cat. # Q33265). Long read sequencing was performed by Plasmidsaurus (Eugene, OR) or SeqCenter (Pittsburgh, PA), using V14 ligation-based library prep (Oxford Nanopore Technologies, Cat. # SQK-LSK114) and R10.4.1 flow cells (Oxford Nanopore Technologies, Cat. # FLO-PRO114M) on the PromethION device. Short-read genome sequencing was performed by SeqCenter using the Illumina tagmentation DNA prep kit (Illumina) and sequenced on a Novaseq X instrument (Illumina) performing a 2 × 151 bp run, using custom 10 bp indices. Demultiplexing, adapter trimming, and quality control were performed using bcl-convert v4.1.5 (Illumina).

Reference genomes were constructed using long nanopore reads by assembly using Flye (89) and consensus polishing using Medaka (Oxford Nanopore). These genomes were subjected to further short-read polishing using PolyPolish (90) and annotation using Bakta (31) via the Bakta web tool. Additional assembly was performed by two methods, hybrid assembly in Unicycler (91) followed by Circlator (92), and hybrid assembly in Trycycler (93), to attempt to detect episomes that may have been excluded from the Flye assembly, but none were detected. Prophages were predicted using PhiSPY (32), supplying the PHROG database (33) for Hidden Markov Model comparison (Data S4). Evolved mutants were sequenced using Illumina short read sequencing as described above, and resulting reads were compared with reference using BREseq (94). Visualization of genomes was performed using ProkSEE (95) via the Proksee web tool, including analysis using the CARD RGI, CRISPR/Cas Finder, Alien Hunter, mobileOG-db, Phigaro and Virsorter, and FastANI (34) plugins. Annotated genomes were routinely viewed using Geneious Prime software (Dotmatics). Genomes were scanned for biosynthetic clusters using antiSMASH 7.0 (30) (Data S5).

### Phylogenetic analysis

Taxonomic classifications of genomes and phylogenetic trees were constructed using GTDB-Tk (96) classify workflow (default parameters) using the Genome Taxonomy Database (GTDB) release 214. Genomes not present in GTDB but shown in the tree (e.g., other model cyanobacterial species) were downloaded from either NCBI or the Pasteur Culture Collection website (https://webext.pasteur.fr/cyanobacteria/).

### Culturing and growth rate measurement

To measure the exponential growth rate in liquid, a Multicultivator instrument with light upgrade was used (MC-2500-OD, Photon Systems Instruments). In this system, air and CO_2_ were mixed using a Gas Mixing System (GMS-150, Photon Systems Instruments), and this mixture was first humidified by bubbling through water, then sparged into the cultures using a 5 × 210 mm porosity B glass filter stick (Ace Glass).

Liquid cultures were prepared in shake flasks as described above, then inoculated into 50 mL of relevant media in Multicultivator vials at an OD_720_ of 0.1, as measured in cuvette using a Nanodrop 2000 (Thermo Scientific). These cultures were first acclimated for 1 hour at 100 µE light at growth temperature; then, the light was increased, and OD measurement was initiated. Unless otherwise stated, cultures were grown in BG-11 medium with 4 µg/L Vitamin-B12 (to facilitate comparison with B-12 auxotrophs), at 37°C, 0.5% CO_2_ flowing at 0.1 L/minute into each vial, and 500 µE of light. Growth rate µ was inferred by fitting an exponential curve to optical density values measured by the Multicultivator between 0.1 and 0.35 and interpreting the slope of the fit using a custom R script (see Fig. S6A for representative fits). Doubling time was calculated as ln(2)/µ.

### High-density batch growth

High-density batch growth cultures were grown in MAD2 medium prepared as per Włodarczyk et al. (18), and modified by substitution of 10 mM Tris-HCl pH 8.0 for 10 mM of TES-KOH pH 8.2. Some precipitation is observed in MAD2 medium. Two microliters of Antifoam 204 were added to each flask as in Włodarczyk et al., but as a 20% solution in 70% ethanol to improve accuracy when pipetting. Patches of relevant strains on solid medium were inoculated into 10 mL AD7 medium and incubated for ~20 hours as above. The resulting culture was used to inoculate 50 mL of MAD2 medium to an OD720 of 0.1 in a 250 mL baffled shake flask fitted with a sponge top and plastic closure. Cultures were incubated as indicated, with the 200 µE condition in an Infors-HT incubator and the 750 µE condition in a Percival incubator. For the 750 µE condition, only 200 µE of light was used for the first 24 hours of incubation, and 750 µE was used thereafter (as in Włodarczyk et al.). At the conclusion of growth, samples were pelleted in preweighed 50 mL conical tubes. Dry weight was determined after 48-hour lyophilization (Labconco FreeZone). Dry weights are summarized in the text with the mean of all replicates, alongside standard error.

### Microscopy

Light microscopy was performed by imaging a wet mount of liquid culture using a Zeiss Axio Imager Z1. Images were processed using FIJI software. TEM images were processed by Harvard Medical School EM facility. TEM samples were fixed in a solution of 1.25% formaldehyde, 2.5% glutaraldehyde, and 0.03% Picric acid in 0.1M cacodylate buffer, pH 7.4. Fixed samples were stained with Osmium tetroxide and uranyl acetate and then dehydrated using an ethanol series followed by propylene oxide. Samples were infiltrated with a 1:1 mixture of EPON resin (Westlake) with propylene oxide for 16 hours at 4°C, then polymerized in Epon resin for 24 hours at 60°C. Embedded samples were sectioned by standard methods before viewing using a JEOL 1200EX transmission electron microscope.

### Biomass characterization

Biomass composition was characterized at the National Renewable Energy Lab (Golden, CO) as reported previously (97) (Data S6). In brief, an Elementar VarioEL cube CHN analyzer was used to determine the C/H/N content. A multiplication factor of 4.78 was used to estimate the total protein content from N content (98). Samples were subjected to acid hydrolysis and the resulting monomeric sugars were measured with a Carbopac HPAEC-PAD system with PA-1 column. Where sugar was undetectable, a value of 0% was used. In rare cases (Fucose, Arabinose in UTEX 3154) where sugar was detectable, but below the limit of quantification, we imputed the value of the sugar based on an established approach (as utilized by the software package MetaboAnalystR4.0.(99) Specifically, we computed the mean of all non-missing measurements for a given strain and set the missing values equal to one-fifth of this mean. Raw data containing missing values (i.e., without imputation) are present in Data S6.

### Metabolomics

Sample preparation was adapted from Jugder et al. (100). Briefly, 400 µL of culture was harvested by centrifugation, then subjected to extraction in 800 µL 80% methanol for 20 minutes in an ultrasonicator bath (Elmasonic P, Elma) at 20°C. Cell debris was removed by centrifugation, and the supernatant was incubated at −80°C for 16 hours and centrifuged again. The resulting supernatant was dried in a SpeedVac Vacuum Concentrator (Thermo) under vacuum and ambient temperature and then stored at −20°C. Samples were analyzed by the Beth Israel Deaconess Medical Center Mass Spectroscopy Core Facility as per previous studies (100) using a 6500 Qtrap triple quadrupole mass spectrometer (AB/SCIEX), Prominence UFLC HPLC (ShimadzuX), and selected reaction monitoring (SRM) of 298 water-soluble metabolites using MultiQuant v3.0 software (AB/SCIEX). The resulting peak areas (Data S7) were analyzed and visualized using Metaboanalyst.ca, using normalization to median, log10 transformation, and mean-centering functions. For a selection of metabolites, raw peak areas are visualized alongside normalized output in Fig. S11.

### RNA-sequencing and downstream analysis

Ten milliliters of cultures were grown in 50 mL unbaffled flasks, at 37°C and 200 µE light, shaken at 220 RPM in a growth chamber maintaining 0.5% CO_2_ for 16 hours. Four replicate cultures were prepared in either BG11 medium or AD7 medium. RNA was isolated from using the Monarch Total RNA miniprep kit (NEB), using the Tough-to-lyse sample protocol with bead-beating (Tissuelyser LT, Qiagen). Ribosomal RNA depletion and strand-specific RNA-sequencing prep were carried out by Azenta Biosciences, along with 2 × 150 bp paired-end sequencing on a HiSeq instrument (Illumina). Demultiplexing using bcl-convert (Illumina) and 8 bp index pairs resulted in >40 million read pairs for each sample. Sickle (https://github.com/najoshi/sickle) was used in paired-end mode for quality filtering (minimum quality = 35; minimum length = 45) and trim adapters from raw sequencing data. We aligned filtered reads with Bowtie2 (101) (params: --very-sensitive-local) to the open reading frames predicted in our annotated genome for UTEX 3222. DEseq2 (102) processed these raw alignment counts to compute differential abundance and statistical metrics. We report significant findings as those with an adjusted *P*-value of less than 0.05.

### Measuring settling phenotypes and cell physical parameters

Sinking/settling of cells was quantified by filling a polystyrene cuvette (Brand-Tech, Semi-micro 759076) with 1 mL of culture diluted to approximately OD_720_ of 1 with culture medium and measuring OD_720_ over time using a Nanodrop 2000c instrument (Thermo). Because this instrument’s light path is 8.5 mm above the bottom of the cuvette, it provides a measure of the OD_720_ above a pellet that accrues over time. Settling occurred at 20°C, shielded from light.

Buoyant masses of single cells were measured using a Suspended Microchannel Resonator (SMR), where single cells flow through a microfluidic channel inside a vibrating cantilever, and the change in vibrational frequency of the cantilever is measured and converted to buoyant mass (103, 104). In this study, the SMR had a cantilever cross-section of 8 × 8 µm, and the resulting frequency measurements were interpreted by custom Matlab code, as previously demonstrated (105). Volumes of single cells were measured using Multisizer 4 Instrument (Beckman Coulter, also known as Coulter Counter, or CC). The SMR and CC were pre-filled with AD7 medium and measurements were performed at approximately 20°C. Both measurements were calibrated using NIST traceable polystyrene beads (Thermo Scientific, Duke Standards). Cultures for these experiments were grown in AD7 medium with 0.5% CO_2_ and 200 µE light at 37°C, for approximately 16 hours. Triplicate measurements were made across unique cultures and multiple days. Gravitational cell sinking velocities were derived as previously reported (58), using Stokes’ Law. For solving Stokes’ Law, we assumed a spherical cell shape, a medium density of 1.02 g/mL, and a dynamic viscosity of 1.07E-3 Pa*s. Cell radius was calculated from cell volume measurements, and buoyant density was calculated from cell volume and buoyant mass measurements. To minimize the influence that population outliers (e.g., cell aggregates) have on the results, we used the modal buoyant mass and volume values, as determined using probability density functions.

### Transformation attempts of UTEX 3222

UTEX 3222 transformation was attempted by electroporation, natural transformation, and conjugation, using either RSF1010 broad host-range plasmids marked with kanamycin resistance (kanR), pBBR1 kanR plasmids (derived from Addgene #85168), or pCB origin plasmids marked with spectinomycin resistance (specR), derived from Addgene #133765. Electroporation and natural transformation were performed as detailed in previous work with PCC 6803 (61). Conjugation was also attempted as per methods described for UTEX 2973 (60), this time transforming suicide plasmids designed to integrate via homologous recombination at either (i) the mutL locus (ii), a locus encoding putative siderophore production, or (iii) a locus encoding multiple restriction/modification systems, adjacent to rpoD. These plasmids were designed with 1 kilobase of homology on either side of a specR marker sequence, and the relevant RP4 origin of transfer for conjugation. Strains were grown in BG-11 medium as elsewhere in this work for preparation of cultures for transformation and plated on BG-11 medium with relevant antibiotics for selection following transformation.

## Data Availability

Strains are publicly available through the UTEX Culture Collection of Algae (University of Texas, Austin). Raw sequence data and assemblies are available at BioProject PRJNA1033390. Associated genome assemblies are available at NCBI Assembly accessions GCA_035320825.1 and GCF_038630775.1.

## SUPPLEMENTAL MATERIAL

Supplemental Data 1 aem.00841-24-s0001.xlsx Conditions of sites sampled at the Baia di Levante.

Supplemental Data 2 aem.00841-24-s0002.csv Results from a RNA sequencing analysis (presented in Fig. 3).

Supplemental Data 3 aem.00841-24-s0003.csv Cell size measurements from light microscopy analysis.

Supplemental Data 4 aem.00841-24-s0004.xlsx Prophage predictions for UTEX 3221, UTEX 3222, and PCC 10605.

Supplemental Data 5 aem.00841-24-s0005.xlsx Biosynthetic gene cluster predictions from antiSMASH.

Supplemental Data 6 aem.00841-24-s0006.xlsx Biomass composition (CHN and carbohydrates) data for UTEX 3222 and UTEX 3154.

Supplemental Data 7 aem.00841-24-s0007.csv Metabolomics peak areas for UTEX 3222, UTEX 2973, and UTEX 3154 biomass.

Supplemental figures aem.00841-24-s0008.pdf Fig. S1-S12.

ASM does not own the copyrights to Supplemental Material that may be linked to, or accessed through, an article. The authors have granted ASM a non-exclusive, world-wide license to publish the Supplemental Material files. Please contact the corresponding author directly for reuse.
