# Supplementary material for: Cyanobacteria newly isolated from marine volcanic seeps display rapid sinking and robust, high-density growth
Source: Appl Environ Microbiol. 2024 Oct 29;90(11):e00841-24. doi: 10.1128/aem.00841-24 (PMC11577773; doi:10.1128/aem.00841-24)
Supplement: Supplemental figures — Fig. S1-S12. [file aem.00841-24-s0008.pdf]

Supplemental Figures

Cyanobacteria newly isolated from marine volcanic seeps display rapid sinking and robust, high density growth

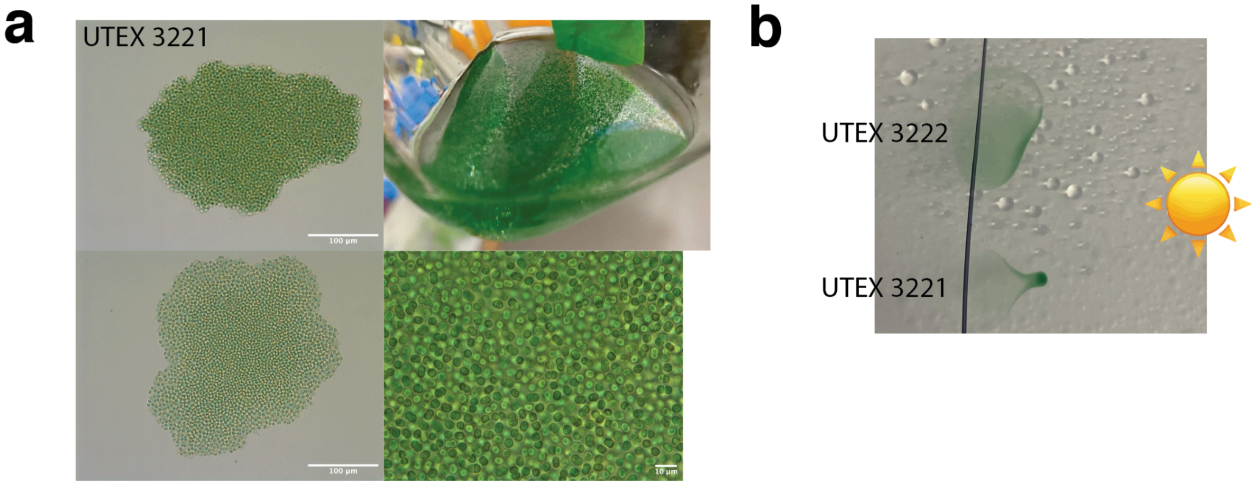

Supplemental Figure 1: a) Micrographs of UTEX3221 aggregates (top, bottom left) and detail of cell packing in an aggregate (bottom right), alongside macroscopic view of aggregates visible when growing UTEX3221 in liquid medium (top right, flask) b) Motility assay comparing UTEX 3221 and UTEX 3222. UTEX 3221 is shown moving toward a light source (direction depicted by sun icon) when lit from the side on 0.3% agarose medium (see methods).

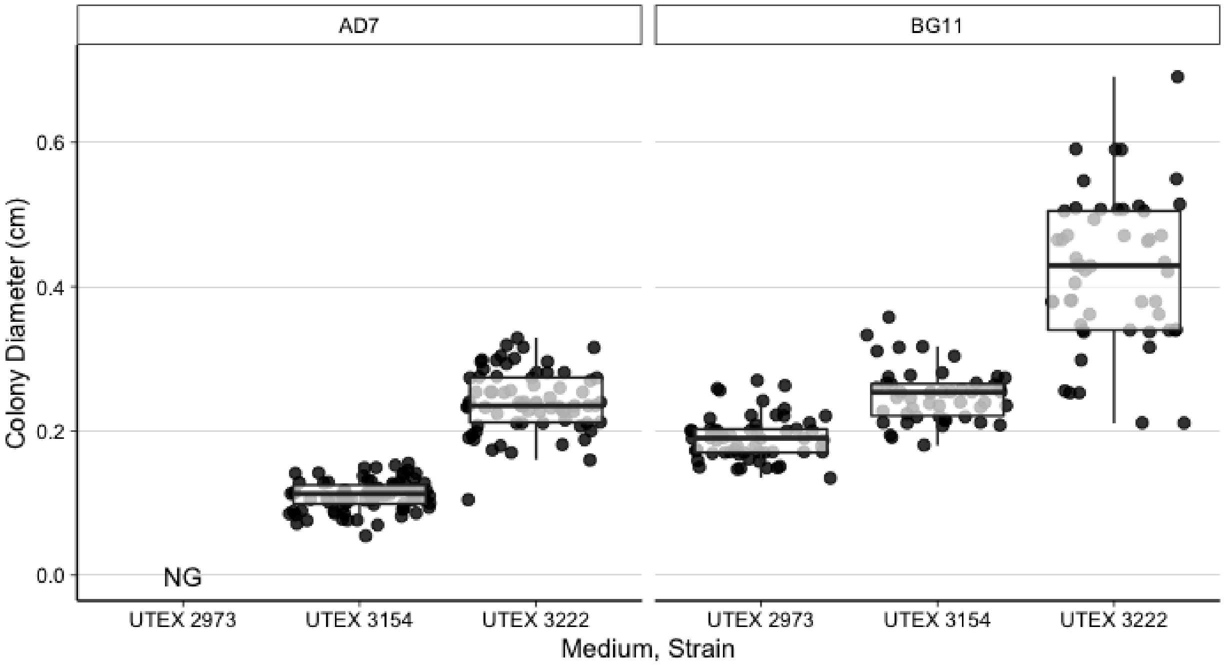

Supplemental Figure 2: Comparison of colony sizes observed in Figure 1B. Measurements of individual colonies are depicted by points, and additionally summarized with box plots in the style of Tukey. No Growth (NG) was observed for UTEX 2973 on AD7 medium. All media additionally supplemented with 4mg/L vitamin B-12 in this experiment.

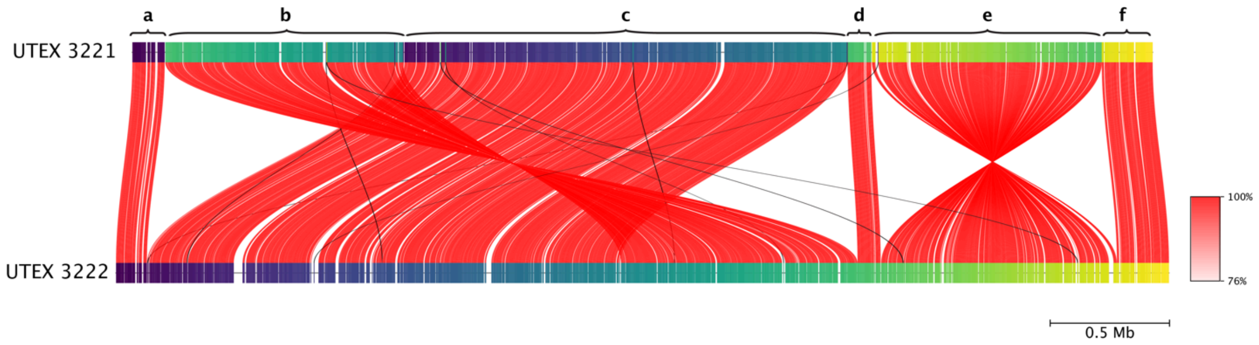

Supplemental Figure 3. A visualization of alignment between UTEX 3222 and UTEX 3221 genomes, generated by Proksee. Red lines indicate reciprocal mapping segments, and are colored by Average Nucleotide Identity. One interpretation is that the strains differ by translocation and inversion of segment b, as well as inversion of segment e.

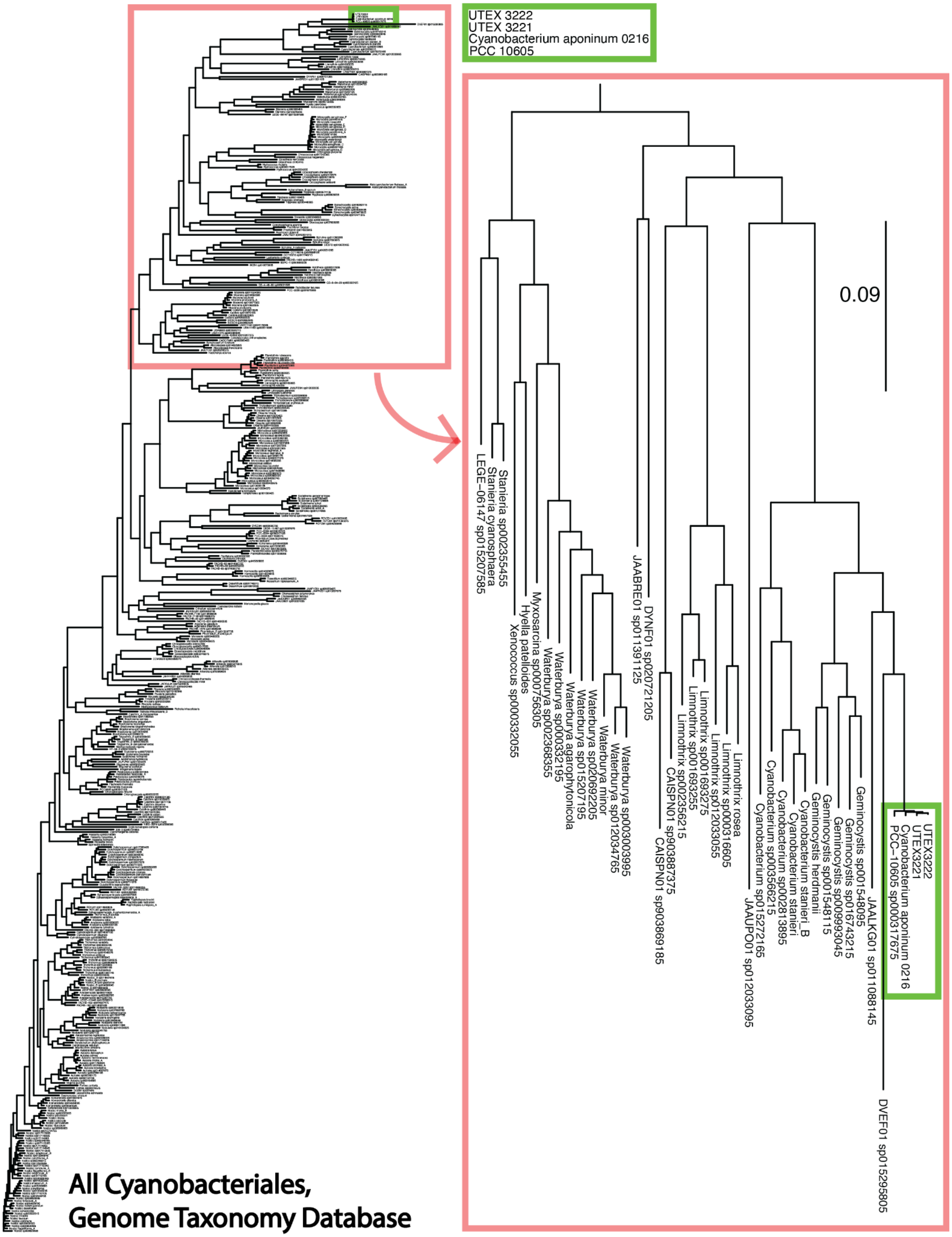

Supplemental Figure 4: Larger tree placing UTEX 3222 and UTEX 3221 in the broader order *Cyanobacteriales*.

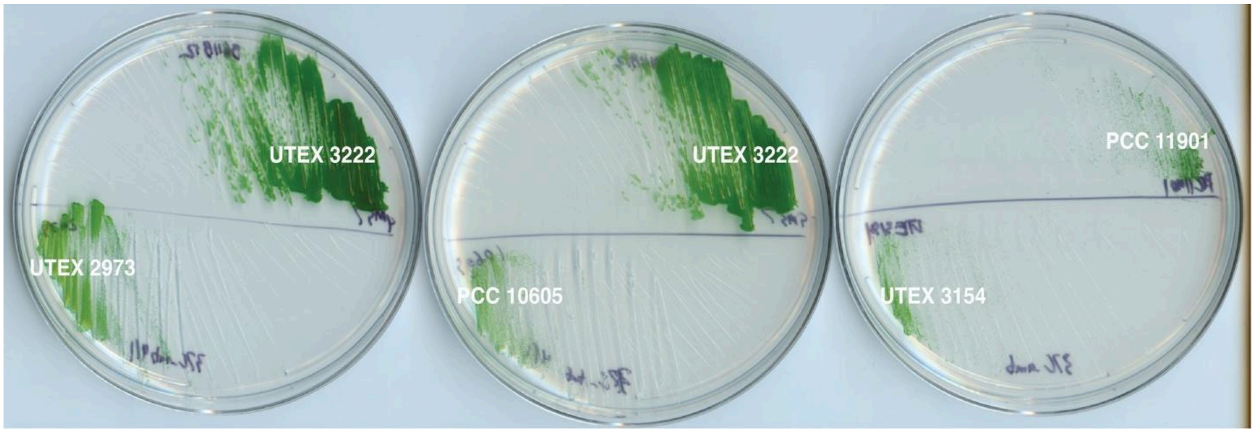

Supplemental Figure 5: A panel of cyanobacteria strains grown on BG11+B12 medium in ambient air conditions, 37C, approximately 100uE light, for 4 days.

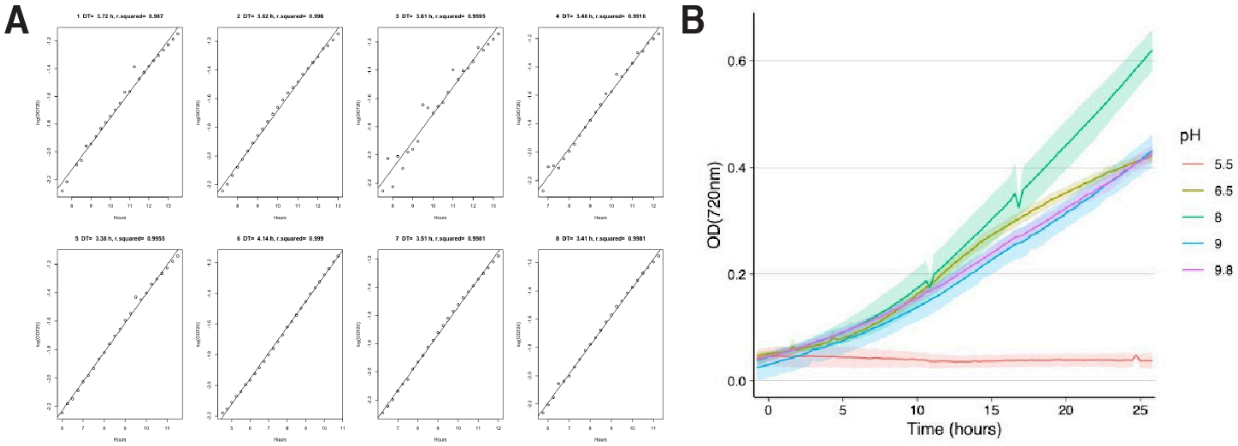

Supplemental Figure 6: Additional information on exponential growth A) Representative linear regressions used to derive exponential growth rate (μ). Individual optical density (OD) measurements are plotted on a logarithmic axis against time. Linear regression is depicted as a line, and doubling times and r^2 values of fits are given. B) Growth curves in varying pH medium. Lines depict the mean value across replicates, and shaded areas depict the standard deviation across replicates.

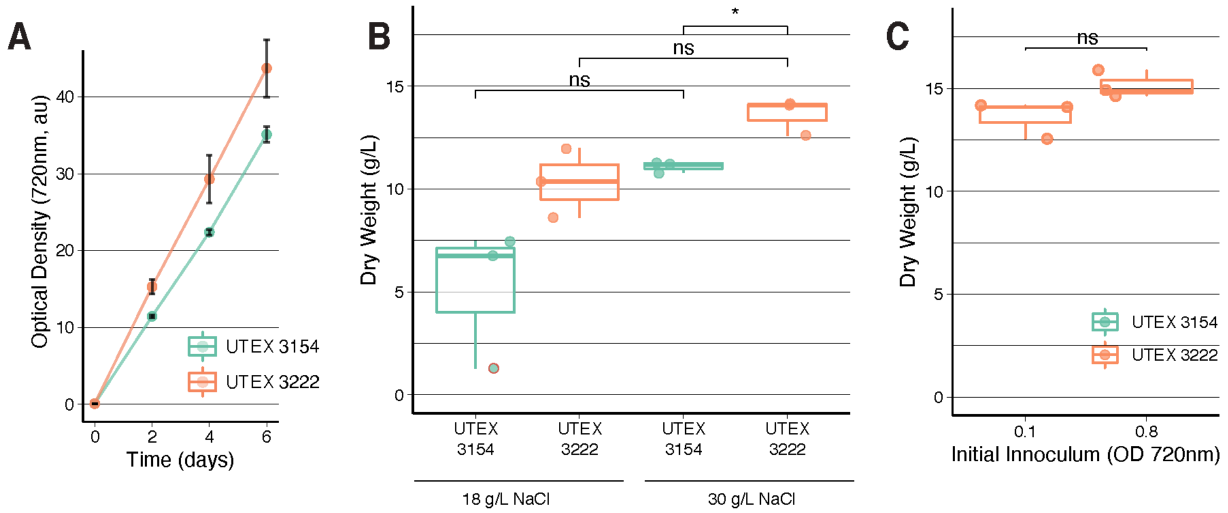

Supplemental Figure 7: Additional High Density Growth A) Optical Density (OD720) monitored over the course of high density batch growth, 0.5% CO2, 200µE light, 37°C, MAD2 medium. Points depict the mean of triplicate measurements, with error bars depicting the Standard Error. B) Comparison of 18g/L NaCl to 30g/L NaCl (MAD2), 0.5% CO_2 and 200µE light, 37°C, 7 days. 18g/L NaCl data are reproduced from Figure 2E for comparison. C) Comparison of inoculation density. Conditions were as above, except with 5% CO2 supplied. Triplicate experiments are depicted by points, and summarized by boxplots. Unpaired t-tests were performed, yielding either non-significant (ns) or $p < 0.05$ (*) results depicted. P-values reading left to right are 0.093, 0.057, and 0.031 for B, and 0.078 for C.

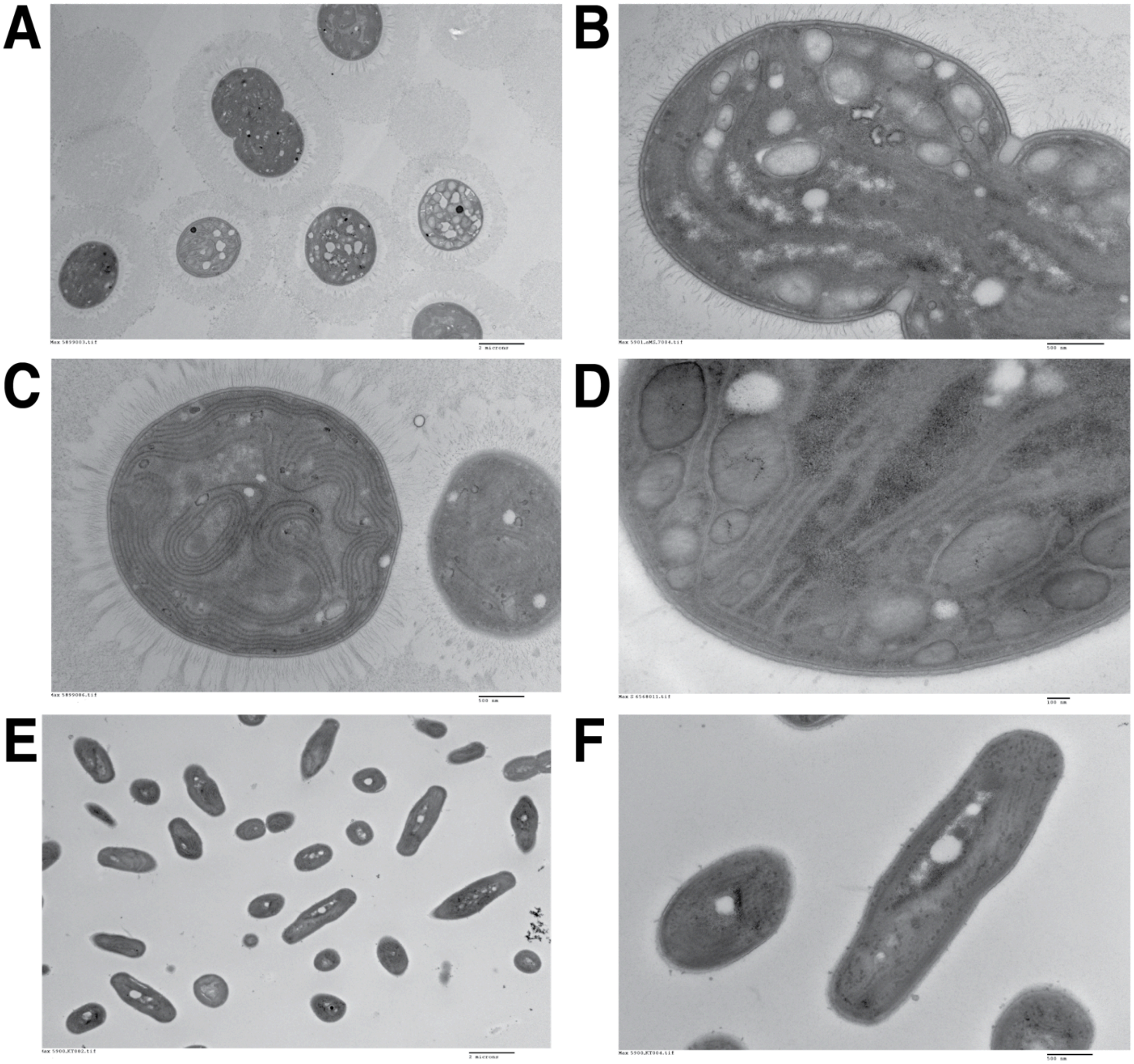

Supplemental Figure 8: Additional featured Transmission Electron Micrographs A) UTEX 3222 at low magnification, showing extracellular material, and a subset of cells featuring putative storage granules B) UTEX 3222, Higher magnification of a dividing cell featuring putative storage granules C) UTEX 3222 with thylakoid membranes and putative pili visible. D) UTEX 3222, higher magnification of a cell with both putative storage granules and thylakoid membranes visible. E) UTEX 3154 for comparison, displaying smaller cells and relative lack of visibly staining extracellular material.

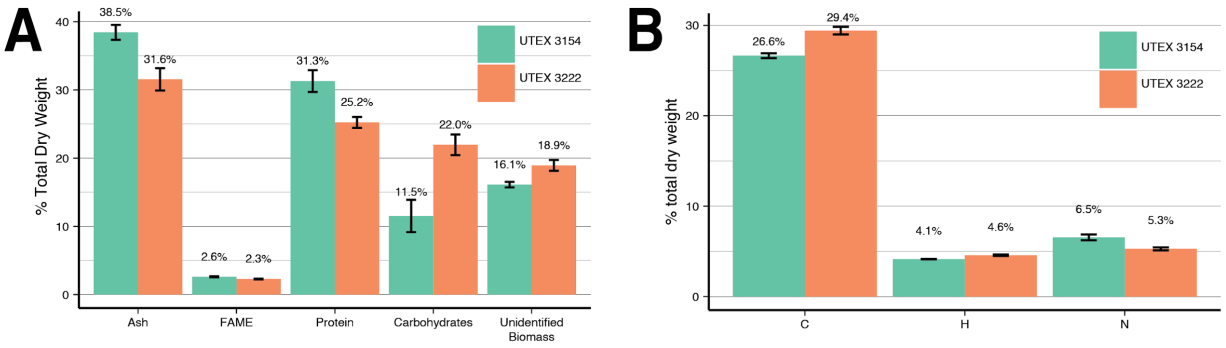

Supplementary Figure 9: further summary of biomass characterization. A) Composition of major macromolecules as a percentage of Total Dry Weight, contrasting with Ash-Free Dry Weight (AFDW) in Figure 4B. B) C/H/N Elemental composition of UTEX 3154 and UTEX3222 biomass. Bars depict the mean of three replicate experiments, and error bars depict the standard error of these measurements.

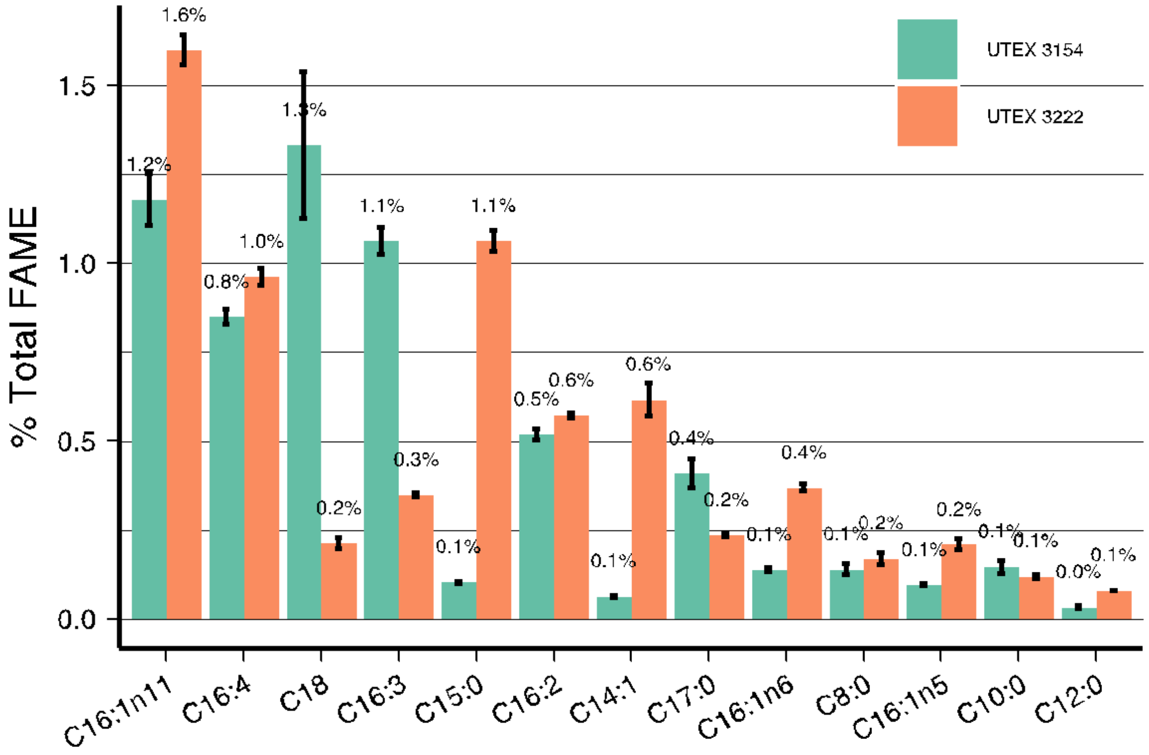

Supplemental Figure 10: comparison of FAME abundance for FAME species, low abundance species not included in Figure 3D.

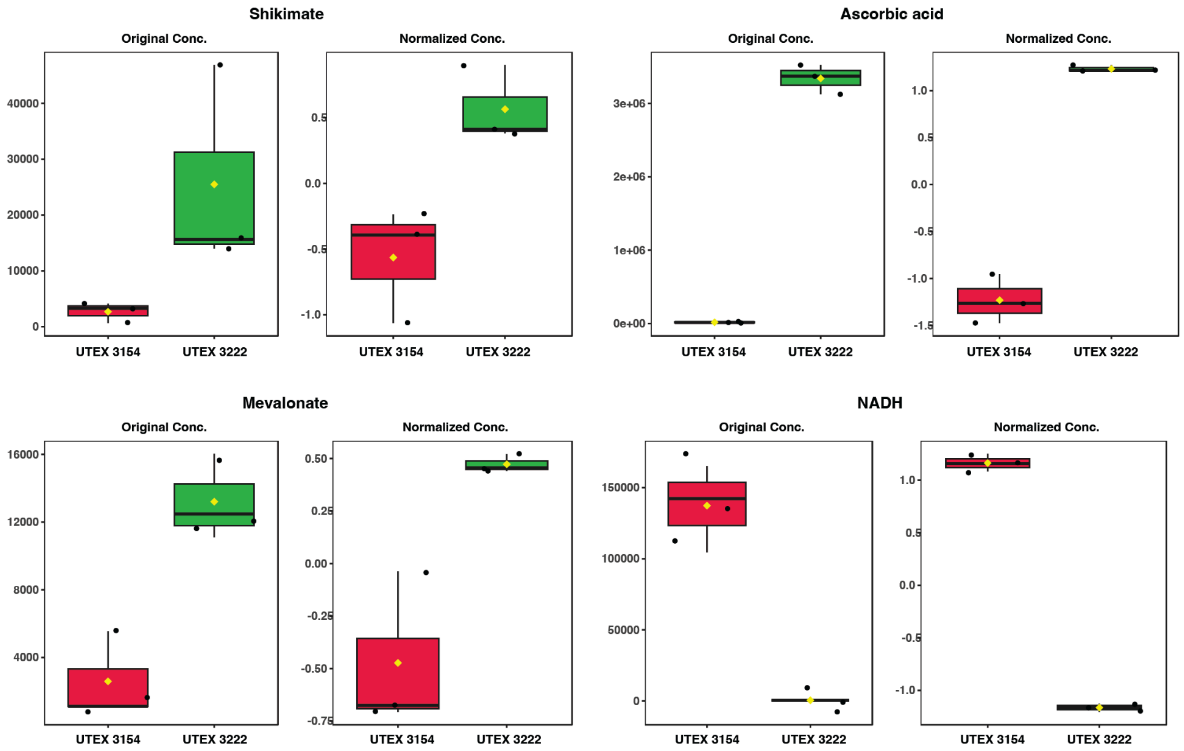

Supplemental Figure 11: Comparison of raw peak area (Original Conc.) to normalized output (Normalized Conc., see Methods for details), for several metabolites of interest.

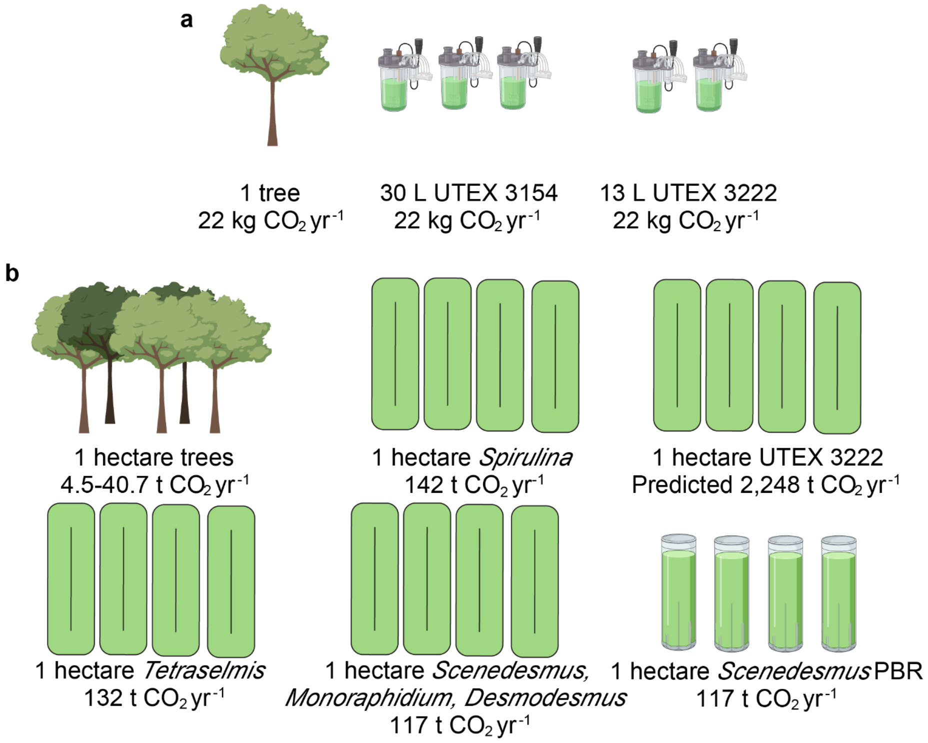

Supplemental Figure 12: A comparison between a single tree and the volumes of cyanobacteria needed to capture the equivalent amount of carbon dioxide. A) Annually, a single tree is approximated to uptake 22 kg CO_2 from the atmosphere [4]. 30 L of UTEX 3154 or only 13 L of faster growing UTEX 3222 would be needed to sequester 22 kg CO yr^{-1}, based on growth rates and carbon concentrations determined in this work, and equations and assumptions used to calculate biofixation rates in previous reports [2]. B) A comparison of the carbon sequestration potential on a hectare of land utilizing trees or different cyanobacteria species. Carbon sequestration numbers were determined using published growth rates in open race-way ponds or a PBR (photobioreactor) as noted. UTEX 3222 areal carbon sequestration rate was extrapolated based on a comparison to the growth rate of *Spirulina*. As is shown, UTEX 3222 may be capable of sequestering 55 times more carbon dioxide than trees and 15 times more carbon dioxide than *Spirulina* on an areal basis.
